# Comparative Transcriptome Profiling of Dairy Goat MicroRNAs from Dry Period and Peak Lactation Mammary Gland Tissues

**DOI:** 10.1371/journal.pone.0052388

**Published:** 2012-12-26

**Authors:** Zhuanjian Li, Xianyong Lan, Wenjiao Guo, Jiajie Sun, Yongzhen Huang, Jing Wang, Tinghua Huang, Chuozhao Lei, Xingtang Fang, Hong Chen

**Affiliations:** 1 College of Animal Science and Technology, Northwest A&F University, Shaanxi Key Laboratory of Molecular Biology for Agriculture, Yangling, Shaanxi, People’s Republic of China; 2 Institute of Cellular and Molecular Biology, Jiangsu Normal University, Xuzhou, Jiangsu, People’s Republic of China; Sun Yat-sen University, China

## Abstract

**Background:**

MicroRNAs (miRNAs) are small noncoding RNA molecules that serve as important post-transcriptional gene expression regulators by targeting messenger RNAs for post-transcriptional endonucleolytic cleavage or translational inhibition. miRNAs play important roles in many biological processes. Extensive high-throughput sequencing studies of miRNAs have been performed in several animal models. However, little is known about the diversity of these regulatory RNAs in goat (*Capra hircus*), which is one of the most important agricultural animals and the oldest domesticated species raised worldwide. Goats have long been used for their milk, meat, hair (including cashmere), and skins throughout much of the world.

**Results:**

In this study, two small RNA libraries were constructed based on dry period and peak lactation dairy goat mammary gland tissues and sequenced using the Illumina-Solexa high-throughput sequencing technology. A total of 346 conserved and 95 novel miRNAs were identified in the dairy goat. miRNAs expression was confirmed by qRT-PCR in nine tissues and in the mammary gland during different stages of lactation. In addition, several candidate miRNAs that may be involved in mammary gland development and lactation were found by comparing the miRNA expression profiles in different tissues and developmental stages of the mammary gland.

**Conclusions:**

This study reveals the first miRNAs profile related to the biology of the mammary gland in the dairy goat. The characterization of these miRNAs could contribute to a better understanding of the molecular mechanisms of lactation physiology and mammary gland development in the dairy goat.

## Introduction

MicroRNAs (miRNAs) are a class of endogenous, small single-stranded (approximately 18–25 nucleotides (nt)), non-coding RNA molecules that regulate gene expression by promoting the translational repression and/or degradation of target mRNAs by binding to their 3′ untranslated regions (3′ UTRs) [1]–[4]. Since the first miRNA gene, *lin-4* [5], was identified in Caenorhabditis elegans approximately two decades ago, extensive research has been conducted on miRNAs in nearly all animals, plants and even viruses [6]. Increasing evidence suggests that miRNAs play important roles in cell differentiation, proliferation, development, apoptosis, and immune response [7]–[11]. Interestingly, many miRNAs are evolutionarily conserved in related species and are expressed in a tissue and/or stage-specific manner [12]–[15]. Moreover, different miRNAs are expressed at different stages of development in the same tissue [16], [17].

Profiling studies in livestock animals have revealed that many miRNAs play important roles in essential biological processes, such as muscle and organ development, immune response, and metabolism. To date, a large number of porcine miRNAs have been identified; research has mainly focused on skeletal muscle and adipose tissue [18]–[22], the ovary and testis [17], [23], and the pituitary gland [24]. Studies of miRNA expression in chicken suggest that a large and diverse group of miRNAs may be essential to the growth and development of skeletal muscle [25], [26], adipose [26], [27], and embryonic [28]–[30] tissues. Extensive studies of bovine miRNAs have been performed, including analyses of the immune system [31], embryonic [32] and adipose [33] tissues, the mammary gland [34], and testicular and ovarian tissues [35]–[37]. However, little is known about miRNA expression in other ruminants, such as sheep and goats.

The goat (*Capra hircus*) is among the most important livestock animals and the oldest domesticated species raised worldwide. It is also one of the best model organisms for mammary gland bioreactor studies. Goats have long been used for their milk, meat, hair (including cashmere), and skins throughout much of the world. Recently, an miRNA profiling study of the skin and hair follicles of goat and sheep identified the expression of 159 miRNAs by computational and microarray analysis [38]. Of these, 105 skin-expressed miRNAs are conserved in the differentiating hair follicles of the goat and sheep, implicating these miRNAs in mammalian hair follicle growth and development. The miRNA catalog of skeletal muscle was first assessed in sheep with four different CLPG genotypes by high-throughput sequencing, which showed that the miRNAs from the CLPG domain target the ORF of DLK1, thereby causing the trans-inhibition underlying polar overdominance [39]. In addition, Chen et al reported 11 candidate miRNAs that were obtained by bioinformatics based on the conservation of miRNA sequences. All 11 of these miRNAs were detected in the brain and 55 were detected in the liver by RT-PCR analysis [40].

The mammary gland is a dynamic organ that undergoes structural changes during the female reproductive cycle. One important aspect of mammary gland research involves investigating the molecular mechanism of lactation. Studies of miRNA in mammary gland biology have mainly focused on human breast cancer, and functional studies have identified specific miRNAs as tumor suppressors and oncogenes that regulate gene expression by targeting mRNAs in breast cancer [41]. Normal miRNAs expression has been studied in the murine mammary gland at different developmental stages [42], [43] and in the bovine mammary gland [34]. These reports have shown that several miRNAs are involved in the maintenance of mouse mammary epithelial progenitor cells.

The identification and validation of miRNAs in the mammary gland at different developmental stages, however, has been limited. Recently, the miRNAs expressed in goat mammary gland tissues during early lactation were investigated, and 300 conserved miRNAs and 131 novel miRNAs were discovered [44]. Notably, there have been no reports on miRNA expression in the goat mammary gland during the dry period or peak lactation to date, and it is necessary to study the change in miRNA expression during all four stages of mammary gland development.

Therefore, in this study, we used Solexa deep sequencing technology to identify and compare the full repertoire of miRNAs expressed in the caprine mammary gland during the dry period and peak lactation. The data obtained provide useful information about the roles of miRNAs in the biological processes of lactation and the mechanisms of target gene expression and regulation.

## Results

### Construction of Mammary Gland Small RNA Libraries using the Solexa Deep Sequencing Technique

**Figure 1:**
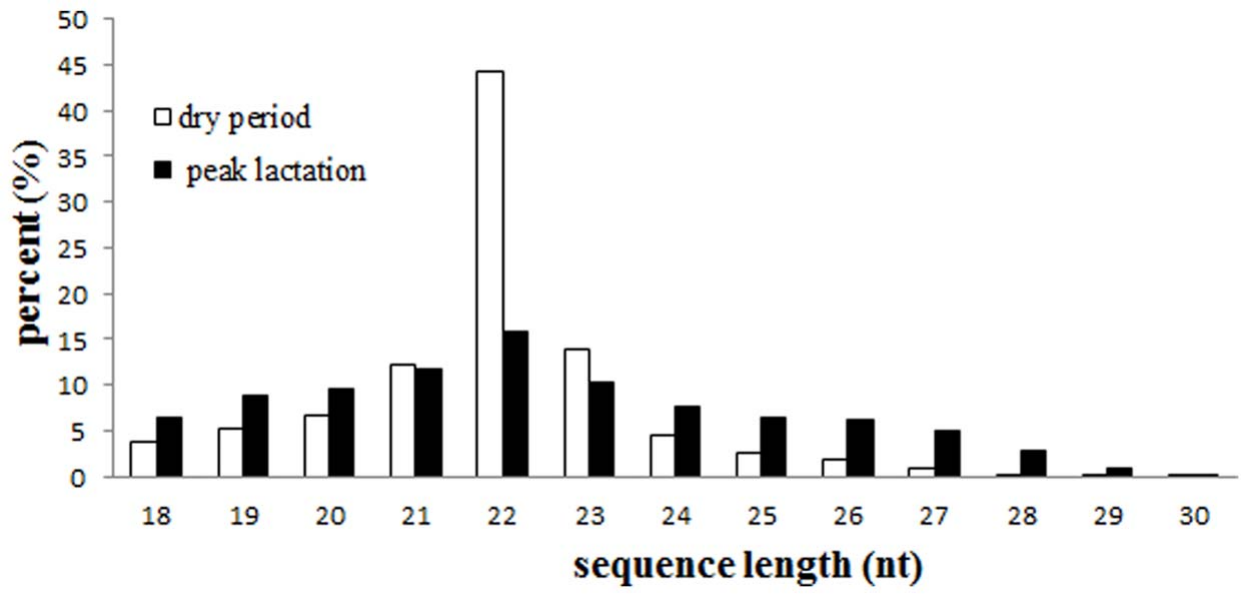
The size distribution of the small RNAs found in the mammary gland during the dry period and peak lactation.

To systematically identify small RNAs and changes in the expression level of miRNAs during mammary gland development, we purified small RNAs from the mammary gland and sequenced them using Solexa high-throughput technology. Mammary gland samples were collected from 2.5-year-old Xinong Saanen Chinese domestic dairy goats. The samples were collected from three animals during the dry period (320 days after lambing, hereafter referred to as the dry period library or D) and peak lactation (75 days after lambing, hereafter referred to as the peak lactation library or P). Total RNA was prepared from these samples, and the RNA from the same developmental stages was then pooled together. Two small RNA libraries were constructed using the small RNA molecules (less than 30 nt) extracted from the D and P mammary gland total RNA. The D and P libraries were sequenced separately using the Solexa deep sequencing technique and generated approximately 15.7 and 17.3 million sequencing reads, respectively. After removing the low-quality reads, adapter contaminant sequences, sequencing reads without insert fragments, reads containing poly(A) stretches and reads of less than 18 nt, a total of 14,851,375 clean reads representing 788,915 unique sequences were recovered from the dry period library, and 15,712,891 clean reads representing 1,108,514 unique sequences were recovered from the peak lactation library (Figure 1 and Table 1). The raw data and processed files for the two libraries have been deposited in NCBI’s Gene Expression Omnibus database (http://www.ncbi.nlm.nih.gov/geo/) under accession number GSE41815.

**Table 1:** Small RNA sequencing statistics for the dry period (D) and peak lactation (P) libraries.

	Dry period library (D)		Peak lactation library (P)	
Category	Total reads (%)	Unique reads (%)	Total reads (%)	Unique reads (%)
raw reads	15706825		17349265	
High quality	15632233 (99.5%)		17220358 (99.3%)	
3′adapter null	15496 (0.10%)		19367 (0.11%)	
Insert null	15085 (0.10%)		10406 (0.06%)	
5′adapter contaminants	78042 (0.50%)		49684 (0.29%)	
Smaller than 18nt	671847 (4.30%)		1427823 (8.23%)	
polyA	388 (0.00%)		187 (0.00%)	
Clean reads	14851375 (94.55%)	788915	15712891 (90.57%)	1108514
Mapped to genome	10615964(67.59%)	227399 (28.82%)	8097817 (46.68%)	246825 (22.27%)

Given that no genomic database and insufficient expressed sequence tag databases are available for caprines. There are full-scale bovine genomic sequences available in Genbank and the goat is a member of the family Bovidae. So, we utilized the bovine genome as a reference for subsequent analysis. The high quality sequences were mapped to the reference genome using SOAP (http://soap.genomics.org.cn). For the selection of the computing algorithm, we chose a tolerance of one mismatch for mapping [45], which resulted in 10,615,964 sequences for the dry period and 8,097,817 sequences for peak lactation being mapped to the reference genome. Next, the small RNAs were classified into different categories according to their annotations. We separated out and discarded rRNA, tRNA, snRNA, scRNA, srpRNA and snoRNA sequences, which were identified using a BLAST against the known noncoding RNAs deposited in the Rfam database and NCBI GenBank databases. Moreover, we aligned small RNA tags to repeat-associated RNA to find matched tags in the sample and to exons and introns of mRNA to find the degraded fragments of mRNA in the small RNA tags (Table S1). The investigation of the unique reads in both the P and D small RNA libraries showed that the largest fractions in both libraries were unannotated small RNAs (unann) (66.29% and 67.44% of total unique clean reads, respectively).

### Identification of Conserved and Novel miRNAs in Caprine

The sequences identified in the two miRNA libraries were first compared with 676 mature miRNAs and 662 precursors in bovine miRBase version 18.0, and 103 mature miRNAs and 55 precursors in sheep miRBase version 18.0 (http://www.mirbase.org/) to discover the conserved caprine miRNAs. A total of 346 conserved miRNAs were identified, and 294 of these were present in both libraries. A total of 303 miRNAs were detected in the P library, and 337 were detected in the D library. These sequences correspond to 5,064,339 (3325 unique sequences) and 8,094,283 (4206 unique sequences) reads (Table 2, Table S2), respectively. These results indicate that the two small RNA libraries encompass almost half the repertoire of the known bovine miRNAs. Notably, many of the known bovine miRNAs were not detected, most likely because those miRNAs are expressed at extremely low levels in the mammary gland or during the specific developmental stages analyzed. In this study, a total of 346 conserved and 95 novel miRNAs were identified in the dairy goat. Using conservative identification criteria, we found that 30 and 4 known sheep miRNAs in miRBase match conserved miRNAs and novel goat miRNAs, respectively (Table S3).

**Table 2:** Summary of conserved miRNAs expressed in the mammary gland during the dry period and peak lactation.

	Total miRNA	miRNA	miRNA*	miRNA-5p	miRNA-3p	A	B	C
D	676	579	42	28	27	662	–	–
Peak lactation	303	256	13	18	16	317	3325	5064339
Dry period	337	284	16	18	19	346	4206	8094283

A the miRNA precursors.

B the unique sRNAs matched to the miRNA precursors.

C the total sRNAs matched to the miRNA precursors.

D known bovine miRNAs in miRBase version 18.0 as a reference.

Sequencing reads that did not match any of the known miRNAs were further analyzed to discover novel miRNAs. miRNAs are derived from miRNA precursors, which have a hairpin-like secondary structure. The novel miRNAs were identified using the Mireap program taking into account of the criteria described by Allen et al [46] and Friedlander et al [47] (see Materials and Methods). Because no caprine genomic sequence is available, the bovine genomic sequence was used as the reference genome for sequence annotation. The sequencing reads that could be mapped to the bovine genome were subjected to a novel miRNA prediction analysis. Sequencing tags that could be mapped to multiple loci in the bovine genome were excluded. To minimize noise, sequencing tags with low abundance (fewer than five reads) were also eliminated. A total of 95 miRNA candidates were identified as having the typical miRNA stem-loop secondary structure, which forms the Dicer enzyme cleavage site. In total, 54 candidates were identified in the P library, 77 in the D library, and 36 candidates in both libraries. (Table S3).

### Experimental Validation of Caprine miRNAs

**Figure 2:**
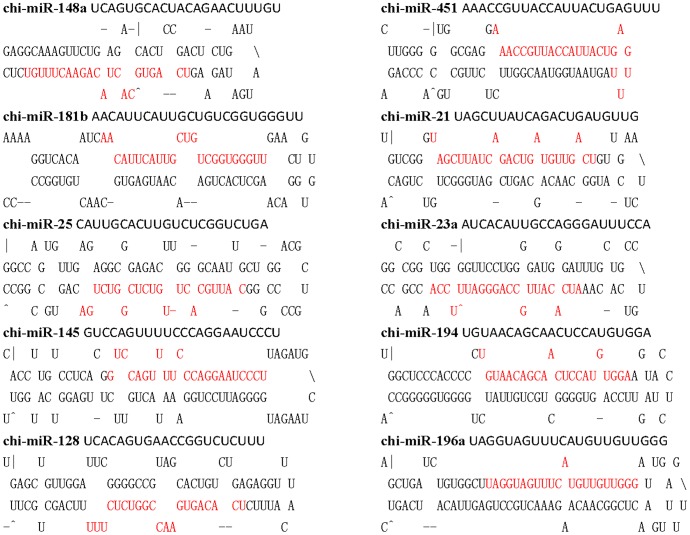
Prediction of the fold-back structure of 10 caprine miRNA precursors. The precursor sequences were obtained by amplifying the corresponding miRNAs gene from goat genomic DNA. The mature miRNA sequences in the precursors are indicated in red.

Because the identification of miRNAs and prediction of novel miRNAs were based on the bovine genome sequences, these sequences may have few sequence differences in the caprine genome. A total of 20 conserved miRNAs were randomly selected and 6 with high expression (>250 sequences in P and D) and 3 specific miRNAs (>100 sequences in P or D but 0 sequences in the other library) were selected for experimental validation (Table S4). The corresponding caprine genomic regions were sequenced in an attempt to predict possible precursor sequences. Primers were designed for PCR amplification of the corresponding miRNA precursor sequences from goat genomic DNA based on the bovine miRNA genomic sequences. The PCR products were sequenced, and the sequences obtained were folded in silico using Mfold software [48] (see Materials and Methods) (Figure 2 and Table 3). These results suggest that these sequences fulfilled the secondary structure criteria for miRNAs. The sequences of the 29 caprine miRNA precursors that could fold into a stem-loop secondary structure were identical to those from cattle (Table S4). These results reliably indicate that that the cloned caprine sequences were conserved miRNAs or novel miRNAs.

**Table 3:** Summary of conserved and putative novel miRNA precursors in the dairy goat.

Name	Length of amplicon (bp)	miRNA sequence (5′-3′)	Length of pre-miRNA/miRNA(nt)	MFE (kcal/mol)	A+U (%)	D/S*
let-7a	483	ugagguaguagguuguauaguu	80/22	−35.6	57.5	S
let-7c	310	ugagguaguagguuguaugguu	84/22	−33.5	52.4	S
let-7d	133	agagguaguagguugcauaguu	87/22	−43.5	50.6	S
let-7i	379	ugagguaguaguuugugcuguu	84/22	−39.6	46.4	S
miR-2478	437	guaucccacuucugacacca	77/20	−20.9	49.4	D
miR-25	613	cauugcacuugucucggucuga	84/22	−38.6	35.7	S
miR-148a	298	ucagugcacuacagaacuuugu	68/22	−28.2	55.9	S
miR-181b	786	aacauucauugcugucgguggguu	110/24	−36.0	51.8	S
miR-145	391	guccaguuuucccaggaaucccu	88/23	−41.6	52.3	S
miR-128	617	ucacagugaaccggucucuuu	81/21	−35.8	50.6	S
miR-199a-3p	564	acaguagucugcacauugguua	104/22	−40.0	46.2	S
miR-451	739	aaaccguuaccauuacugaguuu	71/23	−47.0	50.7	S
miR-93	432	caaagugcuguucgugcaggua	77/22	−36.5	39.0	S
miR-222	1149	agcuacaucuggcuacugggu	110/21	−43.0	51.8	S
miR-21	327	uagcuuaucagacugauguugacu	72/24	−35.8	51.4	S
miR-23a	915	aucacauugccagggauuucca	73/22	−34.0	41.1	S
miR-101	524	uacaguacugugauaacugaa	83/21	−47.3	50.6	S
miR-143	567	ugagaugaagcacuguagcuc	101/21	−48.5	42.6	D
miR-194	693	uguaacagcaacuccaugugga	83/22	−57.6	41.0	S
miR-196a	580	uagguaguuucauguuguuggg	85/22	−45.1	56.5	S
novel_miR_10	557	gaggcgggggucgcucucuuu	83/21	−24.4	48.2	S
novel_miR_12	752	uaauacugccugguaaugaugac	83/23	−34.2	42.2	S
novel_miR_16	569	aucauguaugauacugcaaaca	83/22	−26.0	61.5	S
novel_miR_19	687	agauauugcacgguugaucucu	80/22	−35.2	60.0	S
novel_miR_21	603	gguugaucagagaacauacauu	73/22	−34.9	54.8	S
novel_miR_35	489	gaaaaguucguuuggguuuuc	77/21	−34.2	59.7	D
novel_miR_50	828	caggcuaggagaaaugauugg	78/21	−27.1	68.0	S
novel_miR_51	843	uagcagcgggaacaguacugcag	83/23	−43.7	38.6	S
novel_miR_56	500	uacagugaccaggugacgacg	80/21	−31.5	53.8	S

* In the “Difference” column, “Difference” indicates that the precursor sequence differs between the bovine and caprine sequence (D: different, S: same). The miR-2478 has a G/A mismatch at the 11^th^ nt between the bovine and caprine sequences; the miR-143 precursor has a G/A mismatch at the 97^th^ nt between the bovine and caprine sequences.

### Caprine Mammary Gland miRNA Expression Profiles

Solexa deep sequencing is a useful technique for estimating the expression profile of miRNA genes by measuring sequence frequencies. That is, the sequencing frequencies of the miRNAs in the library can be used as an indicator of the relative abundance of the miRNAs. The numbers of sequencing reads of the miRNAs in the P and D libraries are presented in Table S5. Reads from the intersection of P and D are shown in Table S6∶93.87% of the total small RNAs belong to both P and D; only 4.01% belong to P alone and 2.12% to D alone (Table S6).

In the Solexa deepseq library, more than 13 million sequences were annotated as miRNAs. The sequence reads for the miRNAs are a valuable resource in building the miRNA expression profile of the caprine mammary gland. Some miRNAs, such as let-7a, −7f, −7g, −7c, −7b, miR-103, −107, −1, −143, −423-5p, −29a, −101, −140, −21, −320, −30a–5p, −148a and −199a-3p, were dominantly expressed, with more than 12 million sequences in both the P and D libraries (Table S5). These miRNAs constituted 91.2% of the total conserved miRNA sequences. These results suggest that these miRNAs are abundantly expressed in mammary gland, and might play an important role in regulating the development of this organ. Furthermore, in the P and D libraries, except for miR-151*, miR-126* and miR-455*, the expression levels of miRNA*s (miR-424*, −99a*, −142*, −7a*, −2411* and −21*) were much lower than those of their corresponding miRNAs (Table S5). These results are consistent with a previous report that the miRNA* strands are often degraded rapidly during the biogenesis of the mature miRNAs [49].

### miRNAs with Differential Expression between the Dry Period and Peak Lactation in the Mammary Gland

To gain insight into possible stage-dependent roles of miRNAs during the development of the dairy goat mammary gland, it is interesting to investigate the differential expression profiles of the goat mammary gland miRNAs. Notably, the small RNA length distribution and abundance in the two libraries is different (Figure 1). In the D library, 70.14% of small RNAs are 21–23 nt long, while only 37.75% of the total reads in the P library are 21–23 nt. The 22-nt long RNA molecules were the most abundant small RNAs in both the D and P libraries. Interestingly, the abundance of 22-nt long small RNAs was significantly greater in the D library than in the P library (44.23% and 15.92%, respectively, Figure 1).

Moreover, the number of unique sequences in the peak lactation library was more than 1.4-fold (1108514/788915) higher than that in the dry period library, even though the total numbers of clean reads in both libraries were nearly the same (approximately 15 million). Furthermore, the number of P-specific sequences (903895 reads) was much higher than the number of D-specific sequences (584296 reads) (Table S6). These results indicate that the overall small RNA composition in the two physiological stages is different. The same phenomenon was also observed in raw milk [16], porcine testes [17], and mouse spermatogenesis [50]. The common sequences of the total small RNAs between the two libraries were much higher (93.87%), while the common sequences of unique small RNAs between the two libraries were much lower (12.09%), which indicates that the number of P-specific and D-specific sequences was much smaller than that of the sequences common to both libraries.

Several miRNAs were significantly differentially expressed between the P and D libraries (Figure S1). The identification of miRNAs that were differentially expressed between the two libraries was performed after their numbers were normalized to transcripts per million (see Materials and Methods). The results show that a total of 169 miRNAs were differentially expressed between the P and D libraries, of which 165 were down-regulated and 8 were up-regulated in the mammary gland during peak lactation compared to the dry period (Table S5). In other words, the miRNA concentration in the mammary gland decreased during peak lactation compared to the dry period. These results suggest that the mammary gland miRNAs composition is dynamically altered during the different periods of lactation.

Fifteen differentially expressed miRNAs were selected from the P and D libraries (Table 4). Of these 15 differentially expressed miRNAs, three miRNAs (miR-2887, −451 and −2478) were identified as highly expressed (sequence number>1500 and fold change>1.0) in the peak lactation mammary gland tissue. The most differentially expressed miRNA was miR-2887, which had a 2.04-fold increase in the peak lactation mammary gland tissue, suggesting that this miRNA may play a role in regulating dairy goat lactation. The remaining miRNAs (miR-199b, −128, −25, −145, −98, −222, −181b, −199a-3p, −93, −221, let-7b and let-7c) were expressed at a higher level (sequence number>1500 and fold change>2.0) in the dry period mammary gland tissue. Furthermore, it is essential to identify candidate miRNAs that are present only in the P library or D library. Three candidate miRNAs (>100 sequences in P or D but 0 sequences in the other library) were P or D specific. Interestingly, all 3 candidate miRNAs are novel miRNAs. These results indicate that these miRNAs are involved in regulating dairy goat mammary gland development and/or lactation.

**Table 4:** Abundance and differential expression of the highly abundant caprine miRNAs in the mammary gland.

miRNA name	D	P	D-NE	P-NE	Fold-change log_2_ P-NE/D-NE	*P*-value	Sig-label
miR-2887	385	1671	25.9235	106.3458	2.0364	4.3E−175	**
miR-451	749	1665	50.433	105.964	1.0711	1.25E−68	**
miR-2478	869	1888	58.5131	120.1561	1.0381	8.41E−74	**
miR-199b	1565	290	105.3774	18.4562	−2.5134	3E−227	**
miR-128	8512	1408	573.1456	89.608	−2.6772	0	**
miR-25	36578	5728	2462.937	364.5414	−2.7563	0	**
miR-145	41213	6254	2775.029	398.0171	−2.8016	0	**
miR-98	2531	370	170.4219	23.5475	−2.8555	0	**
miR-222	5223	734	351.6846	46.7132	−2.9124	0	**
miR-181b	5330	734	358.8893	46.7132	−2.9416	0	**
miR-199a-3p	152691	20829	10281.27	1325.6	−2.9553	0	**
miR-93	3593	460	241.9305	29.2753	−3.0468	0	**
miR-221	7848	790	528.4359	50.2772	−3.3938	0	**
let-7b	443620	32993	29870.63	2099.741	−3.8304	0	**
let-7c	341112	27377	22968.38	1742.327	−3.7206	0	**

P and P-NE represent the actual sequencing count and normalized expression level of miRNAs in the small RNA library generated from the mammary gland during peak lactation, respectively.

D and D-NE represent the actual sequencing count and normalized expression level of miRNAs in the small RNA library generated from the mammary gland during the dry period, respectively.

Fold-change [log_2_ (P-NE/D-NE)] indicates the fold change of the miRNAs in a pair of samples.

The *P*-value reflects the significance of the miRNA differential between the samples. A smaller *P*-value indicates a more significant difference in the miRNA level between samples.

A sig-label of ** indicates a fold-change (log2)>1 or (log2)<-1 and a *P*-value<0.01.

### Identification of Caprine miRNAs Expression Patterns via Stem-loop RT-PCR

To validate the miRNA expression level changes and gain insight into the possible roles of miRNAs at different developmental stages or in different tissues/organs in the goat, we conducted stem-loop quantitative real-time PCR [51]. We selected the above-mentioned differentially expressed miRNAs, including the miRNAs that were up-regulated during peak lactation (miR-2887, −451, −2478), the miRNAs that were down-regulated during peak lactation (−199b, −128, −25, −145, −98, −222, −181b, −199a−3p, −93, and −221), and 6 with high expression (>250 sequences in P and D) (novel_mir_10, 12, 19, 21, 50 and 51) and 3 specific miRNAs (>100 sequences in P or D but 0 sequences in the other library) (novel_mir_16, 35 and 56), that were examined by the stem loop RT-PCR. The let-7 family miRNAs were not measured because these miRNAs are well known to be ubiquitously expressed. We investigated the expression patterns of these miRNAs in the skeletal muscle (Mu), heart (He), liver (Li), kidney (Ki), spleen (Sp), lung (Lu), inner fat (Fa), ovary (Ov) and mammary gland at different developmental stages (30 days after lambing (DAL), 75 DAL, 200 DAL, and 320 DAL).

The results indicate that the expression levels of miR-2887, −451 and −2478 were up-regulated in the dairy goat mammary gland during peak lactation. These results were validated by stem-loop RT-PCR. These miRNAs had the lowest expression level at 320 DAL (dry period) and gradually decreased in abundance during the four different developmental stages of the mammary gland. The expression patterns of these miRNAs were similar: high expression in the mammary gland, heart and muscle tissue and low expression in the liver, lung, spleen, kidney, ovary and fat.

**Figure 3:**
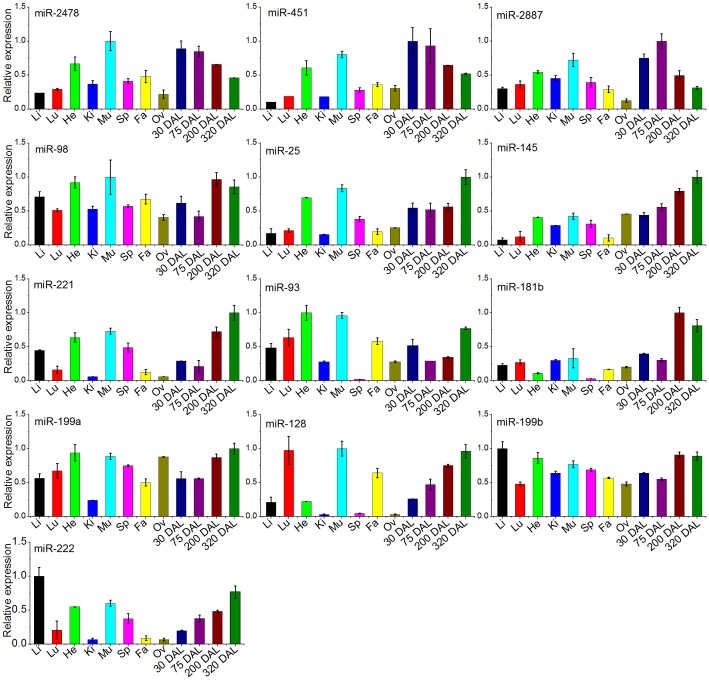
qRT-PCR validation and expression analysis of conserved miRNAs in the dairy goat. Error bars represent one standard deviation of three different biological replicates.

Among the miRNAs that were up-regulated in the dry period mammary gland tissue, several were highly expressed in other tissues: miR-25, −221/222, and −93 in the heart and muscle; miR-222 in the liver; miR-93 in the heart and fat; and miR-128 in the lung and muscle. Furthermore, miR-145 and miR-181b were expressed at low levels in all tissues except for the mammary gland. In contrast, miR-98 and miR-199a/199b were highly expressed in all tissues. A comparison of the expression levels of these miRNAs among different developmental stages of the mammary gland indicated that their expression levels were higher at 200 and 320 DAL than at 30 and 75 DAL. Overall, the results of the qPCR validated the sequencing results. Moreover, the expression of miR-145, −222 and −128 gradually increased over the four developmental stages (Figure 3). These observations indicate that some of the differentially expressed miRNAs underwent dynamic changes in expression during the different stages. These results are consistent with the changes in lactation, showing that these miRNAs may play an important role in the regulation of lactation.

A comparison of the expression profiles of the novel miRNAs among the different tissues revealed that the expression of novel_miR-12 is tissue specific. Other miRNAs were found to be highly expressed in particular tissues, namely novel_miR-10, novel_miR-16, novel_miR-35, novel_miR-50 and novel_miR-56 in the liver and muscle, and novel_miR-19 and novel_miR-21 in the muscle. In contrast, novel_miR-21 was expressed at low levels in all tissue except for the ovary and mammary gland. Furthermore, novel_miR-10, −12, −19 and −21 were expressed at high levels during the different stages. Interestingly, novel_miR-16 and novel_miR-35 were weakly expressed in the dry period mammary gland tissue, and novel_miR-56 was expressed at high levels at 200 and 320 DAL and extremely low levels at 30 and 75 DAL (Figure S2).

## Discussion

In the past several years, miRNAs have been studied extensively. However few studies have focused on the goat, one of the most important livestock animals grown worldwide. Recently, several miRNAs were identified in the goat [38], [40], [44]. However, the identification of most goat miRNAs and their functions, especially in the mammary gland, remains quite limited. A previous study identified 300 conserved miRNAs and 131 novel miRNAs in mammary gland tissues during early lactation [44], but did not investigate miRNA expression in dry period or peak lactation mammary gland tissues. Furthermore, the mammary gland is a dynamic organ whose structure changes throughout the female reproductive cycle, so clarifying the molecular mechanism of lactation is of great importance to the cultivation of dairy goats.

In our study, using high-throughput Solexa technology and a bioinformatics analysis, 303 conserved and 54 novel miRNAs were detected in a library generated from the mammary gland at peak lactation, and 337 conserved and 77 novel miRNAs were detected in a library generated from the mammary gland during the dry period. The majority of the small RNAs were 21–23 nt in length. Most of the sequences were 22 nt in length, which is the typical size range of Dicer-derived products. These results are consistent with the typical small RNA distribution of mammals, such as cattle [16], [34], sheep [39], and pigs [18], [21]. The identification of caprine miRNAs will be useful for studying the role of miRNAs in goat, which is an agriculturally important species and the best model organism for mammary gland bioreactor studies.

Eight members of the let-7 gene family, including let-7a, let-7b, let-7c, let-7d, let-7e, let-7f, let-7g, and let-7i, were sequenced at a high frequency in the mammary gland tissue. In particular, the let-7f was the most abundant miRNA in the two libraries. Sequences from the let-7 family accounted for 41.11% of all clean reads in the dry period mammary gland tissue library and 25.44% of all clean reads in the peak lactation tissue library (Table S5). These data were similar to those from other studies of miRNAs in different tissues [20], [35], [52]. Roush et al [53] also reported that the let-7 family is highly expressed and conserved across animal species, including mammals, flies, worms and plants. These data show that let-7 miRNAs are some of the most important miRNA regulators of fundamental biological processes.

In our study, a total of 346 conserved and 95 novel miRNAs were identified in the dairy goat. Ji et al (2012) detected 300 conserved miRNAs and 131 novel miRNAs from early lactation mammary gland. We detected 95 novel miRNAs in our libraries because we used the bovine miRBase version 18.0 as reference, while Ji et al selecting the bovine miRBase version 17.0. Although most of the miRNAs in our study had been reported previously, different candidate miRNA were detected at different stages. By using high-throughput sequencing technology, the detection capacity of miRNA expression could be on the order of up to 10^6^ sequences. This difference may be due to the fact that samples were obtained from different breeds and different developmental stages, suggesting that some of the miRNAs were expressed in specific temporal patterns [54].

Among the top 20 most abundant miRNAs, 18 miRNAs were present in both samples (Table S5), although their abundance differed significantly between samples. We compared our deep sequencing data with previously reported deep sequencing data, from cattle testis and ovary tissue [35], pig immature, mature [17], backfat [20], pituitary [24], subcutaneous fat and skeletal muscle tissues [21], chicken embryos [30] and caprine skin [38]. We found that 6 miRNAs, including miR-21, −30a, −101, −103/107 and −148a, were among the top 20 most abundant miRNAs in the above-mentioned tissues or organs (Table S7). Moreover, seven of these miRNAs, let-7a, let-7b, let-7c, let-7f, miR-21, miR-29a and miR-143, were detected in both goat and sheep. Among the top 20 most abundant miRNAs, 12 miRNAs were present in the early lactation, peak lactation and dry period mammary gland samples (Table S7), and six of them, miR-1, miR-29a, miR-140, miR-320, miR-199a-3p and miR-2284x were detected in our library. These findings suggest that these miRNAs were expressed in temporally dependent patterns at different developmental stages [54].

The let-7 family was not investigated because these miRNAs are well known to be ubiquitously expressed. Interestingly, the mature sequences of each of these miRNAs were identical among goat, cattle, pigs, chickens, humans, and mice (Figure S3), showing that these mature miRNA sequences are strongly conserved throughout evolution in vertebrates. These data suggest that these miRNAs have a multi-regulatory role in different species. For example, miR-21 is a small multi-faceted RNA that plays an extensive regulatory role in female physiology [35], the uterus [55], adipose tissue [56], skeletal myoblasts [57], immune reactions [35] and almost all forms of cancer [58]. In addition, as one of the top 20 most abundant miRNAs, miR-101 controls mammary gland development by regulating cyclooxygenase-2 expression [59] and the alpha-casein gene [60]. Additionally, miR-21, −101, −103 and −143 are more abundantly expressed in the bovine mammary gland [34], which suggests that these miRNAs may play an important role in the development or physiology of this tissue. Interestingly, the 13 of the top 20 most abundant miRNAs (let-7a, -7b, 7c, 7f, 7g, miR-21, −30a, −103, −107, −143, −148a, −320 and −423-5p) in the mammary gland also appear in the top 20 most abundant miRNAs in raw milk, which shows that these miRNAs in milk originate from the cells of the mammary gland.

Furthermore, 4 of the top 20 most abundant miRNAs (miR-29a, −30a, −101, and −146b) are immune-related miRNAs. miR-29a suppresses immune responses to intracellular pathogens by targeting interferon-γ [61], and miR-146b targets NF-κB signaling as a negative regulator of the innate immune response [62]. In T-cell function, miR-101 is extremely important in the post-transcriptional modulation of Icos, and miR-30 is modulated upon interferon-β exposure [63]. We found that the majority of miRNAs, especially immune-related miRNAs, were expressed at similar levels in the mammary gland and milk. Thus, our results, together with those of others [16], [64]–[66], suggest that the immune-related miRNAs in the milk may be actively secreted by the mammary gland and transferred into the infant’s body via the digestive tract, where they could play a key role in the development of the immune system in infants. Milk is no longer considered to be simply a source of nutrients; rather, it is thought to play a comprehensive role in regulating the immune system as well as infant growth and development.

Other mammary gland related miRNAs in mammals, including miR-126, −99a/99b, −17/92 and−200a, have also been identified in the dairy goat mammary gland. miR-126 regulates progesterone receptors and is involved in development and lactation in the mouse mammary gland [67]; miR-99a/99b modulates transforming growth factor-β-induced epithelial to mesenchymal plasticity in normal murine mammary gland cells [68]; the miR-17/92 cluster is under the control of the key mammary transcription factor STAT5 [69]; miR-200a regulates silent information regulator 1 expression and epithelial to mesenchymal transition-like transformation in mammary epithelial cells [70]; and miR-181a and miR-155 may induce B-cell differentiation [65]. However, whether these miRNAs influence the development of the dairy goat mammary gland by targeting similar gene requires further investigation.

In this study, miRNA expression patterns were profiled in nine different tissues. Although there were diverse expression profiles in different tissues, most miRNAs were ubiquitously expressed. In addition, the comparison of miRNA expression across the four developmental stages of the mammary gland revealed that several miRNAs exhibited a gradual increase (such as miR-451 and −2478) or decrease (such as miR-145, −222 and −128), indicating that these miRNAs may play important regulatory roles in the dairy goat mammary gland.

In conclusion, not only did we identify 441 miRNAs in the caprine mammary gland, including 346 conserved and 95 novel miRNAs, but we also discovered that some miRNAs are differentially expressed among different tissues or physiological stages of the mammary gland. In addition, the comparison of miRNA expression profiles among different tissues and developmental stages of the mammary gland indicated that several potential miRNAs might influence mammary gland development and lactation. However, whether the potential miRNAs actually regulate mammary gland development and lactation should be further investigated. This study provides the first miRNAs profiling study of the caprine mammary gland. The characterization of these miRNAs could facilitate a better understanding of the molecular mechanisms of caprine lactation and mammary gland development.

## Materials and Methods

### Animal Collection and RNA Extraction

The experimental animals used in this study were of a famous Chinese domestic breed of dairy goat known as Xinong Saanen. The dairy goat tissues, including liver, heart, skeletal muscle, lung, inner fat, ovary, spleen and kidney, were collected from three 2.5-year-old Xinong Saanen dairy goats. The mammary glands were collected from three Xinong Saanen dairy goats at different stages: 30 days after lambing (DAL), 75 DAL, 200 DAL, and 320 DAL. All samples were immediately frozen in liquid nitrogen and stored at −80°C. Three samples were then pooled, and the total RNA was isolated from each pooled sample using the Trizol reagent (Takara, Dalian, China) according to the manufacturer’s protocol. The RNA quality and quantity were determined using an Agilent 2100 Bioanalyzer (Agilent Technologies, Palo Alto, CA). All animal protocols were approved by the Review Committee for the Use of Animal Subjects of Northwest A&F University.

### Construction of Small RNA Libraries and Solexa Sequencing

Two miRNA libraries were constructed, namely, 75 DAL and 320 DAL libraries. Small RNAs (less than 30 nt) were collected from the total RNA pool by 15% denaturing polyacrylamide gel electrophoresis. Next, these small RNAs were ligated to a 5′ adaptor and 3′ adaptor and reverse transcribed using the complimentary sequence of the 3′ adaptor. Subsequently, the adaptor sequencing primers were used to amplify the reverse transcription products. Finally, Solexa sequencing technology was used to sequence the small RNAs from the two samples (BGI, Shenzhen China).

### Sequencing Data Analysis and Identification of miRNAs

First, the low quality reads were filtered to remove the reads without the 3′ adaptor, 5′ adaptor-contaminant reads, reads without the insert fragment, reads containing poly(A) stretches, and reads of less than 18 nt. Next, the remaining sequences (clean reads) were mapped to the bovine genome by SOAP (http://soap.genomics.org.cn) with a tolerance of one mismatch. The matched sequences were blasted against the Rfam database (http://www.sanger.ac.uk/software/Rfam) and NCBI GenBank database (http://blast.ncbi.nlm.nih.gov/) to identify and remove the rRNA, tRNA, snRNA, scRNA, srpRNA, and snoRNA sequences. The remaining reads mapped to genomic repeats and to known transcripts (exonic and intronic). All mapping was performed using BLAST with the following parameters: blastall -p blastn, −F F, −e 0.01. Sequences that did not overlap with any annotated sequence were classified as ‘unannotated’. Finally, after being classified into different categories based on sequence similarity and conservation, the remaining reads of our datasets were compared to the currently release of the miRBase to identify miRNAs.

The miRNA identification was performed by comparing the sequenced small RNAs with known bovine and sheep miRNAs in miRBase version 18.0. Potentially novel miRNAs were analyzed in two steps, first using Mireap software and then using Mfold software [48]. The Mireap program, developed by the Beijing Genome Institute (BGI), was used to analyze the structural features of the miRNA precursors to identify all novel miRNA candidates. The resulting structures were retained as novel miRNA candidates only if they met the criteria described by Allen et al [46] and Friedlander et al [47]. Mireap can be accessed at the following link: http://sourceforge.net/projects/mireap/. The novel goat pre-miRNA sequences were checked using Mfold to predict stem-loop structure (http://mfold.rna.albany.edu). The stem-loop hairpins were considered to be typical only when they fulfilled the following criteria: (1) the number of base pairs in a stem was ≥18 nt; (2) the number of errors in one bulge was ≤18; (3) the secondary structures of the hairpins were stable, with a free energy of hybridization under −20 kcal/mol; (4) the percentage of the miRNA in the stem was ≥80%; (5) the length of hairpin (up and down stem+terminal loop) was ≥53 nt; (6) the length of the hairpin loop was ≤22 nt; and (7) the percentage of A and U in the mature miRNA was 30%–70%. If one sequence satisfies these strict criteria, this sequence is considered a candidate of the predicted miRNA precursor.

### Amplification of miRNAs Precursors

The genomic DNA collected from the Xinong dairy goats was isolated from 2% heparin-treated blood samples and stored at −80°C, following standard procedures [71]. Primers were designed by Primer Premier 5.0. The 25 µL PCR reaction volume contained: 50 ng genomic DNA, 0.5 µM of each primer, 1×buffer [including 1.5 mM MgCl_2_], 200 µM dNTPs and 0.625 units of *Taq* DNA polymerase (MBI, Vilnius, Lithuania). The cycling protocol was 5 min at 95°C, 35 cycles of 94°C for 30 s, annealing for 30 s, and 72°C for 40 s, with a final extension at 72°C for 10 min. Amplification products were sub-cloned into the PMD18-T vector (Takara, Dalian, China) and sequenced.

### Analysis of miRNA Expression Levels in the Two Libraries

To compare the miRNA expression levels between two samples to determine the differentially expressed miRNAs, the expression of the miRNAs in two samples (dry period and peak lactation caprine mammary gland tissues) was normalized to obtain the expression in transcripts per million. In cases when the number of transcripts of an miRNA was zero in one of the two libraries, the zero was changed to 0.01 for the comparative analysis; if the number of transcripts of an miRNA was less than 1 in both of the libraries after normalization, this miRNA was discarded during the comparative analysis.

Next, the fold-change and P-value for each miRNA were calculated based on the normalized expression using the formulae shown below: Normalization formula: Normalized expression = Actual miRNA count/Total count of clean reads*1000000. Fold-change formula: Fold_change = log2 (peak lactation/dry period).

*P*-value formula:
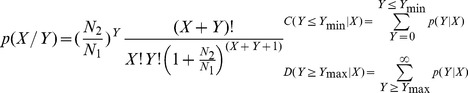
where N1 and X represent the total number of clean reads and normalized expression level of a given miRNA in the small RNA library generated from the mammary gland during the dry period, respectively, and N2 and Y represent the total number of clean reads and normalized expression level of a given miRNA in the small RNA library generated from the mammary gland during peak lactation, respectively.

### Validation of Caprine miRNAs using Stem-loop RT-PCR

Stem-loop qPCR was used to validate the conserved and novel miRNAs according to Chen et al [51]. The real-time PCR was performed using a standard SYBR Green PCR kit (Takara, Dalian, China) in the BioRad CFX96 Real-Time PCR Detection System according to the manufacturer’s instructions. 18S rRNA was used as the reference gene in the qRT-PCR detection of caprine miRNAs, and all reactions were run in triplicate. The relative expression level of the miRNAs was calculated according to the method of Livak and Schmittgen [72]. All of the primers for the RT-PCR and qPCR are shown in the supplementary materials (Table S8).

## Supporting Information

Figure S1 Comparison of miRNAs expression levels in dry period and peak lactation mammary gland tissue. The X and Y axis show expression level of miRNAs in two samples respectively. Red points represent miRNAs with ratio>2; Blue points represent miRNAs with 1/2<ratio<2; Green points represent miRNAs with ratio<1/2; Ratio = normalized expression of the treatment/normalized expression of the control; 1–3 represents peak lactation mammary gland. 2–4 represents dry period mammary gland.
(TIF)

Figure S2 qRT-PCR validation and expression analysis of novel miRNAs in dairy goat. Error bars represent one standard deviation of three different biological replicates.
(TIF)

Figure S3 Comparison of the sequences of the top 20 most abundant miRNAs in model vertebrate animals.
(TIF)

Table S1 The categories of small RNAs detected in the mammary gland during the dry period and peak lactation.
(XLS)

Table S2 Summary of the miRNAs found in the mammary gland during the dry period and peak lactation.
(XLS)

Table S3 Summary of the novel miRNAs found in the mammary gland during the dry period and peak lactation.
(XLS)

Table S4 Summary of stem-loop structures of conserved and novel miRNA precursors in the goat.
(XLS)

Table S5 Comparison of the expression profiles of miRNAs in the caprine mammary gland during the dry period and peak lactation.
(XLS)

Table S6 Summary of the common and stage-specific sequences expressed in P and D.
(XLS)

Table S7 Summary of the top 20 most abundant miRNAs identified by deep sequencing in different livestock animals.
(XLS)

Table S8 The mature miRNA and primer sequences.
(XLS)
