# Supplementary figures and images for: Comparative Transcriptome Profiling of Dairy Goat MicroRNAs from Dry Period and Peak Lactation Mammary Gland Tissues
Source: PLoS One. 2012 Dec 26;7(12):e52388. doi: 10.1371/journal.pone.0052388 (PMC3530564; doi:10.1371/journal.pone.0052388)

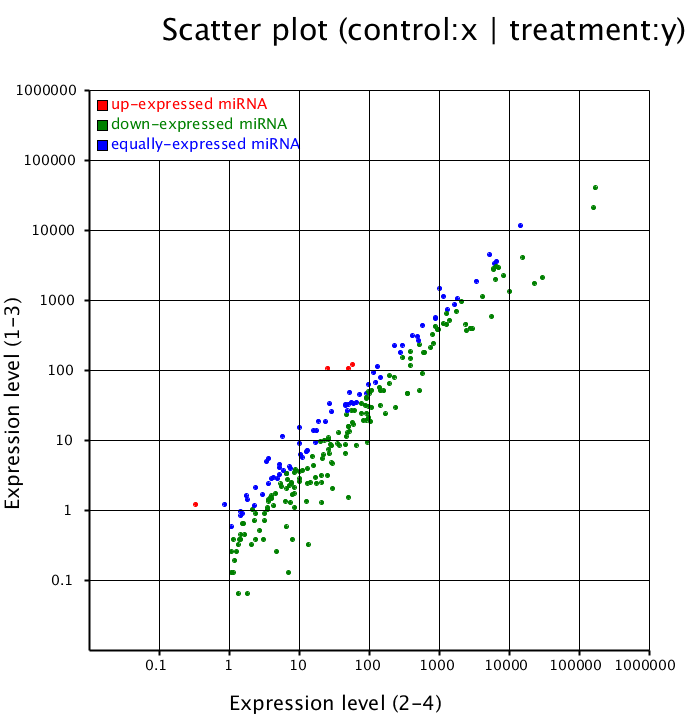

Supplement: Figure S1 — Comparison of miRNAs expression levels in dry period and peak lactation mammary gland tissue. The X and Y axis show expression level of miRNAs in two samples respectively. Red points represent miRNAs with ratio>2; Blue points represent miRNAs with 1/2<ratio<2; Green points represent miRNAs with ratio<1/2; Ratio = normalized expression of the treatment/normalized expression of the control; 1–3 represents peak lactation mammary gland. 2–4 represents dry period mammary gland. (TIF) [file pone.0052388.s001.tif]

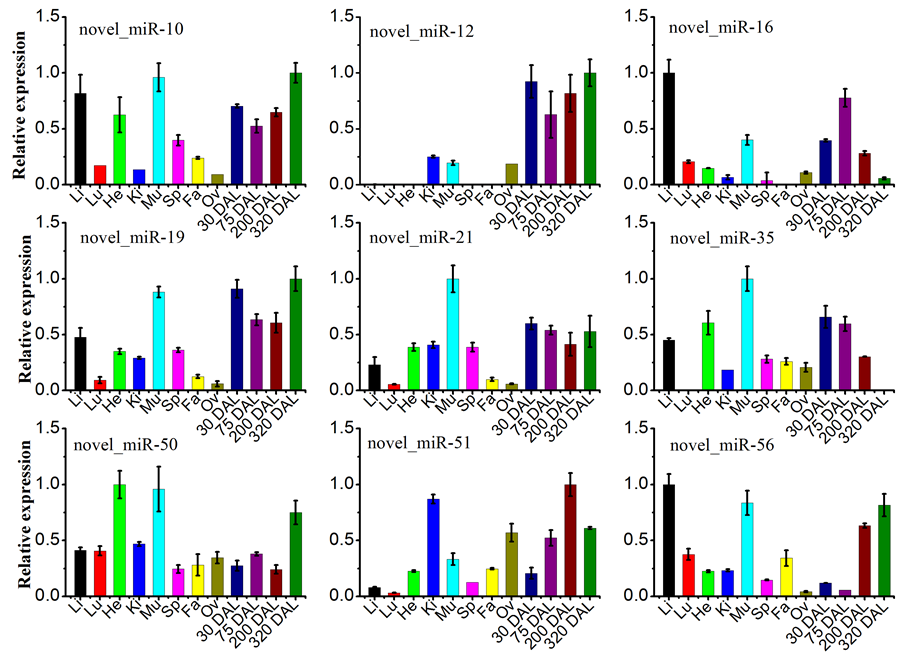

Supplement: Figure S2 — qRT-PCR validation and expression analysis of novel miRNAs in dairy goat. Error bars represent one standard deviation of three different biological replicates. (TIF) [file pone.0052388.s002.tif]

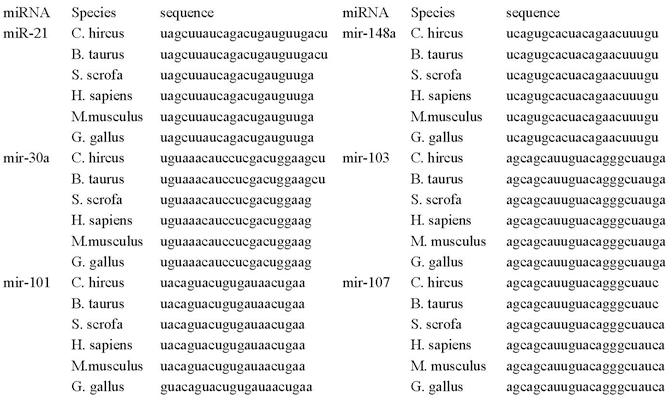

Supplement: Figure S3 — Comparison of the sequences of the top 20 most abundant miRNAs in model vertebrate animals. (TIF) [file pone.0052388.s003.tif]
